# Effects of anti-inflammatory agents on clinical outcomes in people with chronic kidney disease: a systematic review and meta-analysis of randomized control trials

**DOI:** 10.1093/ckj/sfaf001

**Published:** 2025-01-14

**Authors:** Emma R Webster, Adam Perkovic, Brendon L Neuen, Katherine R Tuttle, Vlado Perkovic

**Affiliations:** The Children's Hospital at Westmead, Sydney, NSW, Australia; School of Medicine and Public Health, University of Newcastle, Newcastle, NSW, Australia; Department of Renal Medicine, Royal North Shore Hospital, Sydney, NSW, Australia; The George Institute for Global Health, University of New South Wales, Sydney, NSW, Australia; Nephrology Division, University of Washington School of Medicine, Seattle, WA, USA; Providence Inland Northwest Health, Spokane, WA, USA; University of New South Wales, Sydney, NSW, Australia

**Keywords:** anti-inflammatory therapy, cardiovascular disease, chronic kidney disease, inflammation

## Abstract

**Background:**

Chronic kidney disease (CKD) is characterized by chronic inflammation, which is strongly linked to risk of cardiovascular disease. Anti-inflammatory agents present a novel strategy to reduce the burden of cardiovascular disease in people with CKD, but their effects on clinical outcomes are uncertain.

**Methods:**

A systematic review and meta-analysis was performed to assess the efficacy and safety of anti-inflammatory agents in CKD (PROSPERO CRD42021238755). Medline, Embase and Cochrane databases were searched up to 8 October 2024 for randomized controlled trials of anti-inflammatory agents in CKD with at least 100 patient-years follow-up per treatment arm. The primary study outcome was major adverse cardiovascular events (MACE; defined as myocardial infarction, stroke or cardiovascular death). Other outcomes included CKD progression, malignancy and infection.

**Results:**

Nine trials of 12 042 participants and six different anti-inflammatory classes were identified. Overall, anti-inflammatory agents did not reduce the risk of MACE [risk ratio (RR) 1.01, 95% confidence interval (CI) 0.81–1.24], although there was significant heterogeneity across studies (*P*-heterogeneity = .001; I^2^ = 72%). Anti-inflammatory agents did not have a clear effect on the composite kidney outcome (RR 0.82, 95% CI 0.55–1.22), although there were few events and some trials suggested improvements in the rate of decline in kidney function. Infections were increased with anti-inflammatory agents compared with placebo (RR 1.35, 95% CI 1.01–1.82).

**Conclusion:**

There is currently insufficient evidence to support the use of anti-inflammatory agents to reduce cardiovascular risk or CKD progression in people with CKD, and further dedicated studies in this population are warranted. The potential increased risk of infection with anti-inflammatory agents is an important consideration in the evaluation of these therapies in CKD.

KEY LEARNING POINTS **What was known:**

- Chronic kidney disease (CKD) is an independent risk factor for cardiovascular disease, resulting in a significant morbidity and mortality.
- Systemic inflammation has been implicated in both the progression of kidney disease and pathogenesis of atherosclerosis.
- Some cardiovascular outcome trials have shown improvements in major adverse cardiac events (MACE) with the use of anti-inflammatory therapies such as colchicine and canakinumab; however, the role of these therapies has not be comprehensively explored in people with CKD.

**This study adds:**

- Anti-inflammatory agents had no clear effect on MACE in persons with CKD; however, there was significant heterogeneity across different therapeutic agents, and results were limited by small numbers of trials and data largely derived from subgroup analysis.
- There was insufficient data to reliably examine effects of anti-inflammatory agents on CKD progression.
- Anti-inflammatory agents were associated with increased risk of infection.

**Potential impact:**

- There is currently insufficient evidence to support the use of anti-inflammatory therapy in routine practice to prevent CKD progression or cardiovascular events in persons with CKD.
- These data highlights the limitations of available evidence on the effects of current anti-inflammatory agents in CKD and underscore the role of ongoing dedicated randomized controlled trials in this therapeutic area.

## INTRODUCTION

People with chronic kidney disease (CKD) are at substantially increased risk of cardiovascular disease (CVD) when compared with the general population [1]. The elevated risk is in part due to common underlying risk factors including diabetes, hypertension, and dyslipidaemia [2, 3]. However, a substantial evidence base suggests CKD is an independent CVD risk factor [1], with large meta-analyses finding increased cardiovascular risk and mortality independently associated with both greater levels of albuminuria and lower estimated glomerular filtration rate (eGFR) [4, 5]. Those with advanced CKD experience cardiovascular death at a rate 10–100 times greater than age- and sex-matched peers [6]. Furthermore, in mild to moderate CKD (stages 3A and 3B) the risk of cardiovascular mortality is higher than the risk of progression to kidney failure [4, 5, 7].

Systemic inflammation is implicated in the pathogenesis and progression of atherosclerosis [8, 9]. CKD is also characterized by higher levels of inflammation [10], which may contribute at least partly to the excess burden of CVD in this population [11–13]. Levels of inflammation are highest in people with kidney failure, with large cohort studies indicating that 30%–60% of haemodialysis patients have substantially increased C-reactive protein (CRP) levels [14–17]. Factors such as increased production and reduced kidney clearance of pro-inflammatory cytokines, metabolic acidosis, oxidative stress and exogenous factors such as dialysis membranes have all been implicated in aetiology of this inflammatory state [10]. Additionally, focused research into diabetic kidney disease has implicated innate immune cellular responses alongside inflammatory cytokines ([interleukin (IL)-1, IL-6 and IL-18, and tumour necrosis factor alpha (TNF-α)], chemokines and adhesion molecules in development and progression of the disease [18, 19].

Available evidence suggests inflammation contributes to cardiovascular risk in people with CKD. In haemodialysis patients, elevated baseline CRP levels are associated with increased all-cause and cardiovascular mortality [14, 15, 20]. Similar results are observed in early stages of CKD, with CRP, fibrinogen and advanced-oxidation proteins significantly and independently associated with cardiovascular events [21]. Inflammation may also play a role in the progression of CKD [22, 23].

Emerging evidence from trials in non-CKD populations has found significant cardiovascular benefits associated with the use of anti-inflammatory treatments such as colchicine and canakinumab for the secondary prevention of cardiovascular events [24]. The use of anti-inflammatory agents may thus represent an important strategy to reduce risks of major cardiovascular events and possibly improve kidney outcomes in people with CKD. We therefore conducted a systematic review and meta-analysis to evaluate the effects of anti-inflammatory agents on these clinical outcomes in people with CKD.

## MATERIALS AND METHODS

### Search strategy

This systematic review and meta-analysis was reported according to the Preferred Reporting Items for Systematic Reviews and Meta-Analyses (PRISMA) guidelines [25] and was prospectively registered on PROSPERO (CRD42021238755).

A search was performed of MEDLINE via OVID, EMBASE and the Cochrane Central Register of Controlled Trials (CENTRAL) databases for all randomized controlled trials of anti-inflammatory agents up to 8 October 2024. There were no imposed language or time restrictions, and references of select existing review articles and clinical guidelines were reviewed to further capture relevant randomized trials for inclusion. Two reviewers (E.R.W. and A.P.) independently screened the eligibility of studies for inclusion based on their title and abstract with the full texts of selected studies also reviewed by both authors. Discrepancies between the two authors were resolved through consensus discussion with third author (V.P.).

### Study selection

Studies selected for inclusion were randomized controlled trials of anti-inflammatory agents, including colchicine, methotrexate, IL-1 inhibitors (canakinumab, anakinra, rilonacept), IL-6 inhibitors (tocilizumab, sarilumab, ziltivekimab), JAK-1/2 inhibitors (baricitinib), NRF-2 activators (bardoxolone), ASK-1 inhibitors (selonsertib), PKC-alpha inhibitors (ruboxistaurin), CCL-2 inhibitors (CCX140-B) and sPLA2 inhibitors (darapladib, varespladib). Randomized trials (or CKD subgroups of randomized trials) with <100 patient years follow-up per treatment arm were excluded. Studies were also excluded if the control arm was exposed to anti-inflammatory therapy or if the agent was being used for the treatment of cancer, gestational trophoblastic disease or graft-versus-host disease.

Selected studies were reviewed to identify whether a CKD subgroup was defined and whether relevant outcomes in these participants had been reported. If data were not available, the study authors were contacted to determine whether unpublished data could be obtained.

### Data extraction and quality assessment

Data from included studies was extracted and reviewed by two authors. The primary outcome was a composite of major adverse cardiovascular events [MACE; defined as myocardial infarction (MI), stroke, and cardiovascular death]. Other cardiovascular outcomes included MI, stroke, cardiovascular death, hospitalization for heart failure and death from any cause. Kidney outcomes included change in eGFR; change in albuminuria; and a composite kidney outcome of worsening kidney function (40% or 50% decline in eGFR or doubling of serum creatinine), kidney failure (transplantation or dialysis) or death due to kidney failure. Any serious adverse events, malignancy and infection were also evaluated where data were available. For studies which reported data on CKD subgroups, data relating to both the entire study population and CKD subgroup were extracted.

Assessment of study bias was performed using the Cochrane risk-of-bias tool for randomized trials (RoB 2; Supplementary data, Fig. S1) [26].

### Data analysis

Data analysis was undertaken using Stata, version 18.0 [27]. Treatment effects were quantitatively synthesized using a random effects mode, with treatment estimates expressed as relative risks with associated 95% confidence intervals (95% CI). Hazards ratios (HRs), incidence rate ratios and risk ratios (RRs) (based on the number of events and participants) were pooled in order of preference to maximize information obtained from included trials. Heterogeneity was quantified using the I^2^ test and a summary of effect estimated was determined with a random effects model by means of the DerSimonian and Laird Method. In studies where there were variable doses of study drug, data were pooled where possible to facilitate analysis compared with the placebo arm. Because of variations in the reporting of change in eGFR over time (with different studies reporting mean change, eGFR slope and rates of eGFR decline in active and control arms without treatment effects), change in eGFR was reported descriptively without quantitative synthesis.

## RESULTS

We identified 9186 unique articles through database searches. Following title and abstract screening, we reviewed 478 full texts with 34 studies subsequently assessed for CKD data. The PRISMA flow chart of study selection is displayed in Supplementary data, Fig. S2. Nine studies involving 12 042 participants were included in this review. The included trials evaluated bardoxolone (two studies), colchicine (two studies), darapladib (two studies), canakinumab (one study), CCX140-B (one study) and methotrexate (one study), all compared with placebo [28–38]. People with CKD represented the entire study population in four trials (2834 participants), whilst the remaining five trials included a CKD subgroup analysis from a broader study population (9208 participants). Three studies were determined to have low risk of bias, whilst the remaining six studies had some potential issues. No studies were identified to have high risk of bias (Supplementary data, Fig. S1).

### Cardiovascular outcome

**Figure 1:**
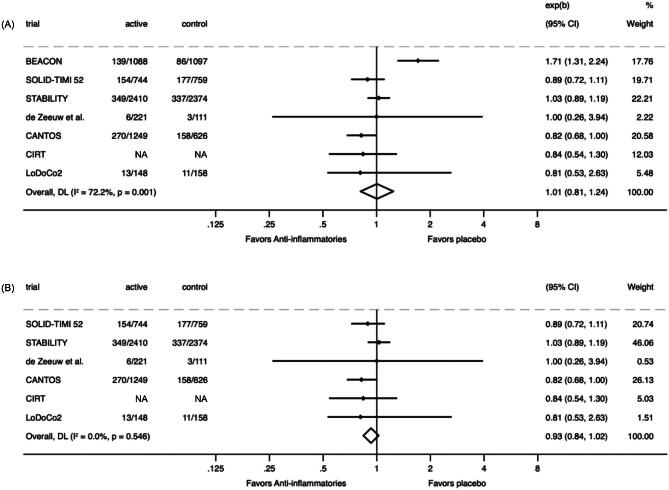
(**A**) the effect of anti-inflammatories on major cardiovascular events in CKD; (**B**) effect of anti-inflammatories on major cardiovascular events in CKD excluding the BEACON trial.

Overall, anti-inflammatory agents did not have an effect on MACE (1703 events; RR 1.01, 95% CI 0.81–1.24; Fig. 1A). However, significant heterogeneity was observed (*P*-heterogeneity = .001; I^2^ = 72.2%), driven by an increased risk of MACE with bardoxolone in the Bardoxolone Methyl Evaluation in Patients with Chronic Kidney Disease and Type 2 Diabetes Mellitus: the Occurrence of Renal Events (BEACON) trial. In a sensitivity analysis excluding BEACON, no clear effect was observed (RR 0.93, 95% CI 0.84–1.02), a lack of effect consistent across the remaining trials (*P*-heterogeneity = .546; I^2^ = 0.0%; Fig. 1B).

**Figure 2:**
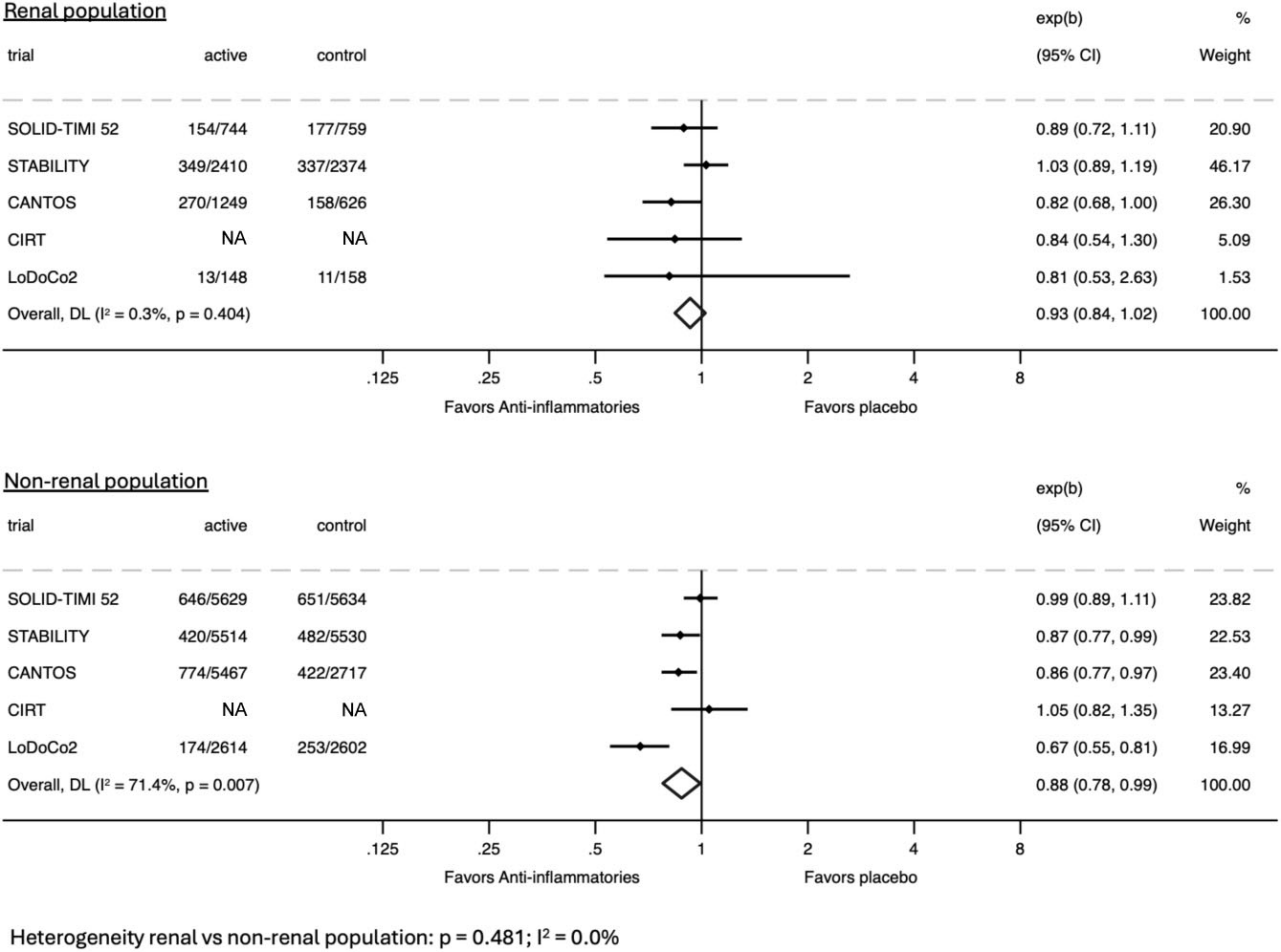
Comparison of the effect of anti-inflammatories on MACE in cardiovascular outcome trials enrolling participants with and without CKD.

Within the MACE outcome, a comparative meta-analysis was performed to evaluate the impact of subgroups within the primary outcome data (Fig. 2). The analysis found that the larger, non-CKD populations of the five trials showed benefit overall in those without CKD (RR 0.88, 95% CI 0.78–0.99) and did not suggest any difference in the effect of anti-inflammatory therapy compared with placebo on MACE from those with CKD (RR 0.93, 95% CI 0.84–1.02, *P*-heterogeneity = .481; I^2^ = 0.0%).

There was generally insufficient data to evaluate effects on other cardiovascular outcomes with data limited to very few trials. Overall, no clear effect was observed on myocardial infarction (2 events; RR 0.50, 95% CI 0.03–7.96; Supplementary data, Table S2), stroke (39 events; RR 1.00, 95% CI 0.53–1.85; Supplementary data, Fig. S3), cardiovascular death (237 events; RR 1.01, 95% CI 0.56–1.79; Supplementary data, Fig. S4) and all-cause mortality (390 events; RR 1.10, 95% CI 0.69–1.74; Supplementary data, Fig. S5).

### Kidney outcomes

Kidney outcomes were reported in less than half of the included trials. Change in eGFR was reported in four trials, with three of these studies (two of bardoxolone and one of methotrexate) showing a statistically significant reduced rate of eGFR decline with anti-inflammatory therapy compared with placebo. The kidney composite outcome was unable to be meta-analysed as no events occurred in the either of the trials of colchicine or CCX140-B, whilst in remaining study of bardoxolone no effect was seen (HR 0.82, 95% CI 0.55–1.24). Thus, there is an absence of evidence for effects on clinical outcomes.

Change in albuminuria was only reported in two studies with contrasting effects observed. The trial of bardoxolone found a statistically significant mean increase in albuminuria of 58.6% (95% CI 50.6–67.0), whilst CCX140-B showed a reduction in albuminuria for both drug doses, with the greatest effect seen within the 5-mg arm (change in urinary albumin-to-creatinine ratio –16%, 95% upper confidence limit –5%; *P* = .01 compared with placebo).

### Adverse outcomes

**Figure 3:**
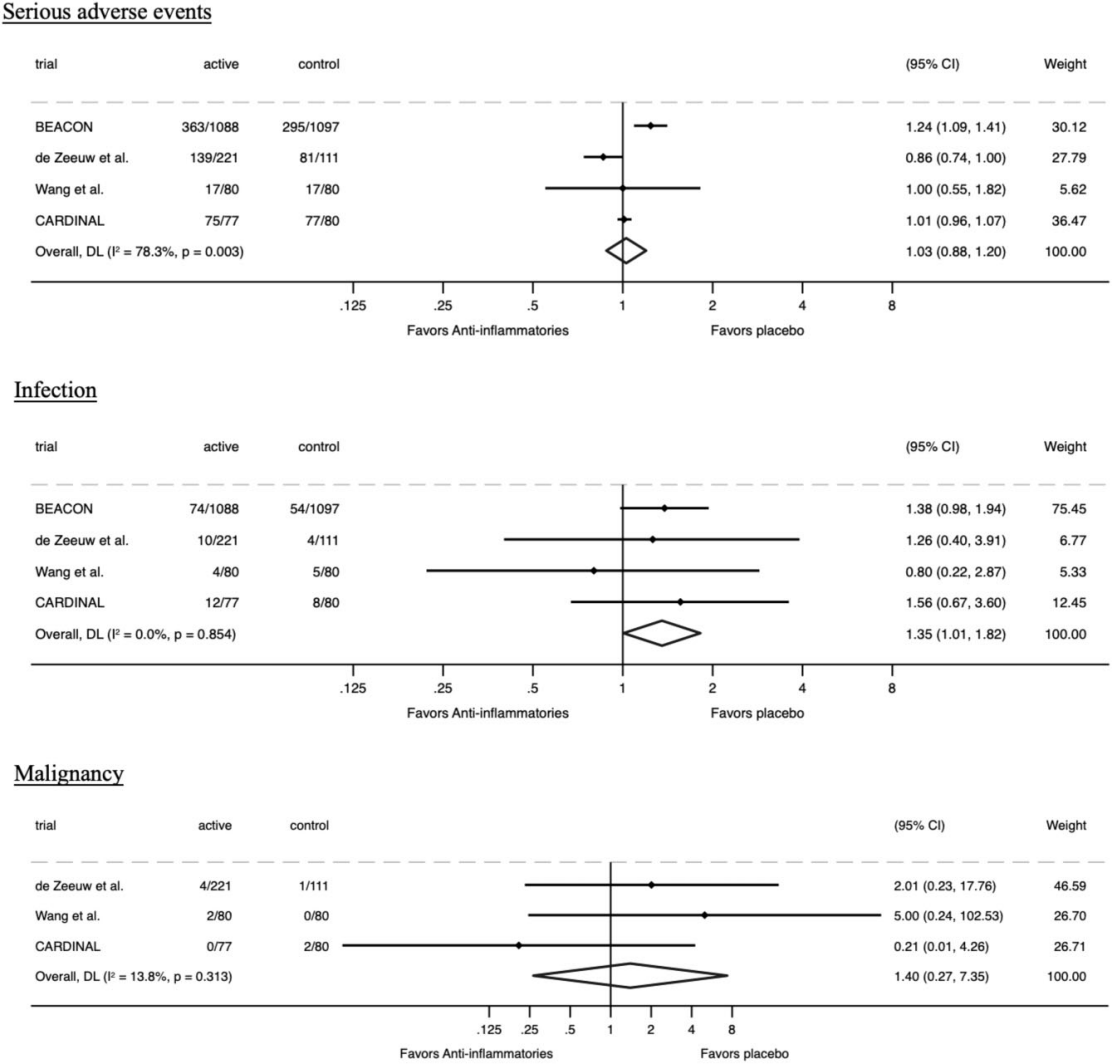
Effect of anti-inflammatories on serious adverse events, infection and malignancy in persons with CKD.

Anti-inflammatory agents did not affect the risks of serious adverse events (RR 1.03, 95% CI 0.88–1.20; Fig. 3). However, the risk of infection was increased with anti-inflammatory agents (RR 1.35, 95% CI 1.01–1.82; Fig. 3). No clear effect was observed on cancer incidence (RR 1.40, 95% CI 0.27–7.35; Fig. 3).

## DISCUSSION

In this systematic review and meta-analysis, we made three main observations. First, we observed no clear effect of anti-inflammatory therapy on MACE in persons with CKD, although there was significant heterogeneity across different agents. For example, while canakinumab appeared likely to reduce MACE, an increased risk was observed with bardoxolone. Second, data on cardiovascular events were limited, with most data derived from subgroups of patients with CKD in cardiovascular outcome trials in the general population. Third, we observed an increased risk of infections with anti-inflammatory agents, an effect that appeared consistent across agents. Taken together, the data highlight the need for large, dedicated trials of anti-inflammatory agents to better understand the efficacy and safety of novel anti-inflammatory agents in persons with CKD.

For the primary outcome of MACE, an important finding was clear heterogeneity in effects on this outcome, driven by bardoxolone in the BEACON trial. In BEACON, bardoxolone increased the risk of MACE, possibly related to volume expansion and increased afterload [28]. This finding contrasts with canakinumab which reduced the risk of MACE overall in The Canakinumab Antiinflammatory Thrombosis Outcome Study (CANTOS) trial (HR 0.88, 95% CI 0.79–0.97) with consistent effects in its CKD subgroup (HR 0.82, 95% CI 0.68–1.00). However, given the limitations of a subgroup analysis and the absence of further trials of IL-1 inhibitors, the implications of these results for people with CKD remain uncertain. Only two trials enrolled an exclusive CKD population and only BEACON studied persons with stage 4 CKD, thus limiting the certainty of evidence in CKD. Additionally, it was difficult to discriminate the efficacy of individual drug classes as only a single trial was available for all but one agent. The absence of data was most notable for colchicine, which has the most robust evidence base of any anti-inflammatory agent for cardiovascular protection in general populations [39], and has now received US Food and Drug Administration approval for the prevention of cardiovascular events in high-risk groups [40]. However, this likely reflects its risk of toxicity in CKD [41], limiting both its use and the possibility of future trials of colchicine in CKD and ultimately emphasizing the need for a unique approach to target inflammation in those with CKD.

Kidney outcomes were inconsistently reported across trials, limiting our ability to draw robust conclusions about the kidney protective effects of anti-inflammatory agents. No effect could be established on kidney composite outcome, due in large part to the low number of events observed. Additionally, almost all kidney outcome data came from trials of bardoxolone. We found an improvement in eGFR slope with bardoxolone, however previous trials of bardoxolone have been unable to distinguish haemodynamic effects on eGFR from a potential protective effect on CKD progression. Therefore, it is unclear whether improvements in eGFR seen with bardoxolone in this review reflect true preservation in kidney function [42]. However, the small but statistically significant improvement in slope of eGFR within the CKD population with methotrexate in the CIRT trial is noteworthy, particularly in context of new data suggesting causality been inflammation and CKD progression [43, 44]. A recent large two sample Mendelian randomization study found that people with risk alleles associated with greater circulating IL-6 levels, had a 24% increased risk of developing end-stage renal disease (odds ratio 1.24, 95% CI 1.01 to 1.52) [43]. Similar studies from Finland assessing association between cytokines and kidney outcomes found 10 inflammatory factors to be causally associated with increased risk of CKD, with high levels of TNF-α particularly associated with rapid eGFR decline [44]. This emerging research coupled with these initial results underscores the importance of ongoing trials powered to establish the effect of anti-inflammatory therapy on CKD progression.

Total adverse events were not increased by anti-inflammatory therapy, however we did observe an increased risk of infections with anti-inflammatory agents compared with placebo. This is particularly relevant as the risk of adverse events such as community-acquired infection increases with declining eGFR [45]. Therefore, this finding highlights the importance of careful monitoring of infection outcomes in future trials of anti-inflammatory agents in CKD and suggests that strategies to mitigate this risk, such as vaccination, may need to be implemented as part of these trials.

A key strength of this work lies in the systematic and comprehensive synthesis of the totality of the randomized evidence addressing this question. Despite this, there were insufficient outcome data leading to significant residual uncertainty about the effects of anti-inflammatory agents in CKD. Specifically, the reliance on data from subgroup analysis meant available results were generally underpowered to demonstrate an effect. Moreover, there were few kidney outcomes outside of trials of bardoxolone. Most CKD study populations included individuals with early-stage CKD with few data on those with advanced CKD including kidney failure. Given both inflammation and the risk of cardiovascular mortality increase with higher CKD stage [11, 46], larger effects may be seen in these groups. However, this absence is likely again a consequence of medication safety concerns given the increased risk of medication related adverse outcomes with advancing CKD stage [47].

Additional studies including the phase II trial RESCUE of ziltivekimab and POSIBIL_6_ESKD of clazakizumab; whilst not meeting size criteria for inclusion in this review; have provided additional insight into the modulation of inflammation with anti-inflammatory therapy in advanced stage CKD [48, 49]. These trials showed sustained, statistically significant reduction in CRP with IL-6 inhibition, however they were underpowered to correlate this with clinical outcomes [48, 49]. Additional insight will come from further trials such as the ZEUS trial which is evaluating the effect of ziltivekimab on a primary outcome of MACE in 6200 patients with CKD and evidence of inflammation and atherosclerotic cardiovascular disease [50]. This will provide important information as to whether anti-inflammatory therapy can safely modify cardiovascular risk in a high-risk CKD population.

## CONCLUSION

There is currently insufficient evidence to support the use of anti-inflammatory agents to reduce cardiovascular risk or CKD progression in people with CKD, and further dedicated studies in this population are warranted. The potential increased risk of infection associated with anti-inflammatory agents is an important consideration in the evaluation of these therapies in CKD.

## Supplementary Material

sfaf001_Supplemental_File

## Data Availability

The data underlying this article are available in the article and in its online supplementary material.
