# Supplementary material for: Effects of anti-inflammatory agents on clinical outcomes in people with chronic kidney disease: a systematic review and meta-analysis of randomized control trials
Source: Clin Kidney J. 2025 Jan 14;18(3):sfaf001. doi: 10.1093/ckj/sfaf001 (PMC11997804; doi:10.1093/ckj/sfaf001)

SUPPLEMENTARY PUBLICATION MATERIAL

Table of Contents

Table S1: Study Characteristics…….………………………………………………………..2

Table S2: Individual Outcomes……………………………………………………………3

Table S3. Search strategy…………………………………………………………………6

Figure S1. Risk of bias assessment…………………………………………………...9

Figure S2. PRISMA diagram……………………………………………………………..10

Figure S3. Stroke..……………………………………………………………………......11

Figure S4. Cardiovascular Death…………………………………………………………12

Figure S5. Mortality…….……………………………………………………..………13

Table S1: Study characteristics.

Study	Location	Population	Intervention	CKD Population Included	Duration (months)	Size of CKD population (total population)
**De Zeeuw 2013 BEACON**	USA, EU, Australia, Canada, Israel, Mexico	Stage 4 CKD, T2DM	Bardoxolone	Stage 4 CKD	9	2185
**De Zeeuw 2015**	Belgium, Czech Republic, Germany, Hungary, Poland, UK	T2DM with proteinuria	CCX140-B	UACR of 100–3000 mg/g + eGFR > 25 m mL/min per 1·73 m²	12	332
**Warady 2022 CARDINAL**	USA, Europe, Japan, Australia	Alport syndrome	Bardoxolone	Stage 1-3 CKD + UACR ≤3500 mg/g	24	157
**Wang 2021**		T2DM, albuminuria	Colchicine	Stage 1-3 CKD + UACR 30-300 mg/g	36	160
**Nidorf 2020 LoDoCo2**	Australia, Netherlands	Coronary disease	Colchicine	Stage 3a CKD (Sub-group)	29	306 (5522)
**Ridker 2017 CANTOS**	39 Countries	Prior MI	Canakinumab	Stage 3 CKD (Sub-group)	48	1875 (10061)
**Ridker 2019 CIRT**	North America	Coronary disease, T2DM	Methotrexate	Stage 3 CKD (Sub-group)	28	740 (4786)
**O'Donoghue 2014 SOLID-TIMI 52**	36 Countries	ACS	Darapladib	Stage 3 CKD (Sub-group)	30	1503 (13026)
**White 2014 STABILITY**	39 Coutrnies	Coronary heart disease	Darapladib	Stage 3 CKD (Sub-group)	44	4784 (15828)

Table S2: Individual outcome results in included studies.

Trial	Anti-inflammatory	Placebo	Hazard Ratio (95% CI)
	no. of events/ no. of patients		
**Myocardial Infarction**			
de Zeeuw et al.	1/221	1/111	0.5 (0.03 - 7.96)
**Stroke**			
BEACON	17/1088	18/ 1097	0.95 (0.49 -1.84)
de Zeeuw et al.	2/221	0/111	
CARDINAL	0/77	1/80	
Wang et al.	1/80	0/80	
**Cardiovascular Death**			
BEACON	27/1088	19/1097	1.44 (0.8 – 2.59)
CANTOS	116/1165	75/ 592	0.79 (0.60 – 1.03)
**Congestive Heart Failure Hospitalisations**			
BEACON	96/ 1088	55/ 1097	1.83 (1.32 – 2.55)
**Renal Composite Outcome**			
BEACON	43/ 1088	51/1097	0.82 (0.55 – 1.24)
de Zeeuw et al.	0/221	0/111	
Wang et al.	0/80	0/80	
**Total Adverse Events**			
BEACON	363/1088	295/1097	1.24 (1.09 – 1.41)
de Zeeuw et al.	139/221	81/111	0.86 (0.74 - 1.00)
CARDINAL	75/77	77/80	1.01 (0.96 – 1.07)
Wang et al.	17/80	17/80	1.00 (0.55 – 1.82)

**Cancer**			
de Zeeuw et al.	4/221	1/111	2.01 (0.23 -17.76)
CARDINAL	0/77	2/80	
Wang et al.	2/80	0/80	
**Infection**			
BEACON	74/1088	54/1097	1.38 (0.98 – 1.94)
de Zeeuw et al.	10/221	4/111	1.26 (0.4 – 3.91)
CARDINAL	12/77	8/80	1.56 (0.67 – 3.60)
Wang et al.	4/80	5/80	0.80 (0.22 – 2.87)
**Death (Any cause)**			
BEACON	44/1088	31/1097	1.47 (0.93 – 2.32)
de Zeeuw et al.	2/221	0/111	
CANTOS	198/1165	115/592	0.87 (0.71 – 1.08)

**Trial**	**Anti-inflammatory**	**Placebo**	**Mean Difference** (95% CI/ p-value)
	Mean Change (95% CI)		
**Change in eGFR** (mL/min/1·73 m²)			
BEACON	5.5 (5.2 to 5.9)	-0.9 (-1.2 to -0.5)	6.4 (5.9 to 6.9)
de Zeeuw et al.			
5 mg dose	–2·4 (–4·4 to –0·4)	–2·6 (–4·6 to –0·6)	P= 0·88
10 mg dose	–3·8 (–5·9 to –1·8)	–2·6 (–4·6 to –0·6)	P= 0.39
CARDINAL	-1.4 (-4.5 to 1.7)	-8.5 (-11.4 to -5.6)	7.1 (2.9 to 11.3)
CIRT			1.58 (0.37 to 2.8)

Change in Albuminuria (%)

BEACON	58.6 (50.6 to 67.0)	1.7 (-3.2 to 6.8)
de Zeeuw et al.		
5 mg dose	–11(20 to –1)	-2 (-11 to 9%)
10 mg dose	-18 (-26 to -8)	-2 (-11 to 9%)

Table S3: search strategy.

Medline and Embase via Ovid	The Cochrane Library
1. Exp Randomized Controlled Trial/<br>2. Exp Random Allocation/<br>3. Exp Single-Blind Method/<br>4. Exp Double-Blind Method/<br>5. (random$ adj5 trial$).tw.<br>6. (random$ adj5 allocation$).tw.<br>7. (Blind$ adj5 method$).tw.<br>8. 1 OR 2 OR 3 OR 4 OR 5 OR 6 OR 7<br>9. Exp Mortality/<br>10. Exp Cardiovascular diseases/<br>11. Exp Heart failure/<br>12. Cardiovascular death.tw.<br>13. Exp Myocardial infarction/<br>14. Exp Stroke/<br>15. Exp Coronary disease/<br>16. Revasculari?ation.tw.<br>17. Cardiovascular outcome$.tw.<br>18. 9 OR 10 OR 11 OR 12 OR 13 OR 14 OR 15 OR 16 OR 17<br>19. Exp Methotrexate/<br>20. Exp Folic Acid Antagonists/<br>21. Exp Colchicine/<br>22. Interleukin 1 inhibit$.tw.<br>23. Interleukin 1 antagonis$.tw.<br>24. IL 1? inhibit$.tw.<br>25. IL 1? antagonis$.tw.<br>26. IL1? inhibit$.tw.<br>27. IL1? antagonis$.tw.<br>28. Canakinumab.tw.<br>29. Anakinra.tw.<br>30. Rilonacept.tw.<br>31. Interleukin 6 inhibit$.tw.<br>32. Interleukin 6 antagonis$.tw.<br>33. IL 6 inhibit$.tw.<br>34. IL 6 antagonis$.tw.<br>35. IL6 inhibit$.tw.<br>36. IL6 antagonis$.tw.<br>37. Toclizumab.tw.<br>38. Sarilumab.tw.<br>39. Satralizumab.tw.<br>40. Siltuximab.tw.<br>41. Ziltivekimab.tw.<br>42. Exp Janus Kinase Inhibitors/	1. Randomized Controlled Trial<br>2. Random Allocation<br>3. Single-Blind Method<br>4. Double-Blind Method<br>5. Random* trial*:ti,ab,kw<br>6. Random* allocation*:ti,ab,kw<br>7. Blind* method*:ti,ab,kw<br>8. 1 OR 2 OR 3 OR 4 OR 5 OR 6 OR 7<br>9. Mortality<br>10. Cardiovascular diseases<br>11. Heart failure<br>12. Cardiovascular death:ti,ab,kw<br>13. Myocardial infarction<br>14. Stroke<br>15. Coronary disease<br>16. Revasculari?ation:ti,ab,kw<br>17. Cardiovascular outcome*:ti,ab,kw<br>18. 9 OR 10 OR 11 OR 12 OR 13 OR 14 OR 15 OR 16 OR 17<br>19. Methotrexate<br>20. Folic Acid Antagonists<br>21. Colchicine<br>22. Interleukin 1 inhibit*:ti,ab,kw<br>23. Interleukin 1 antagonis*:ti,ab,kw<br>24. IL 1? Inhibit*:ti,ab,kw<br>25. IL 1? Antagonis*:ti,ab,kw<br>26. IL1? Inhibit*:ti,ab,kw<br>27. IL1? Antagonis*:ti,ab,kw<br>28. Canakinumab:ti,ab,kw<br>29. Anakinra:ti,ab,kw<br>30. Rilonacept:ti,ab,kw<br>31. Interleukin 6 inhibit*:ti,ab,kw<br>32. Interleukin 6 antagonis*:ti,ab,kw<br>33. IL 6 inhibit*:ti,ab,kw<br>34. IL 6 antagonis*:ti,ab,kw<br>35. IL6 inhibit*:ti,ab,kw<br>36. IL6 antagonis*:ti,ab,kw<br>37. Toclizumab:ti,ab,kw<br>38. Sarilumab:ti,ab,kw<br>39. Satralizumab:ti,ab,kw<br>40. Siltuximab:ti,ab,kw<br>41. Ziltivekimab:ti,ab,kw<br>42. Janus Kinase Inhibitors

43. Baricitinib.tw.<br>44. Tofacitinib.tw.<br>45. Upadacitinib.tw.<br>46. Nuclear factor erythroid 2-related factor 2 activat$.tw.<br>47. NRF 2 activat$.tw.<br>48. NRF2 activat$.tw.<br>49. Bardoxolone.tw.<br>50. ASK 1 inhibit$.tw.<br>51. ASK1 inhibit$.tw.<br>52. ASK 1 antagonis$.tw.<br>53. ASK1 antagonis$.tw.<br>54. Apoptosis signal-regulating kinase 1 inhibit$.tw.<br>55. Apoptosis signal-regulating kinase 1 antagonis$.tw.<br>56. Selonsertib.tw.<br>57. Protein kinase C alpha inhibit$.tw.<br>58. Protein kinase C alpha antagonis$.tw.<br>59. PKC-alpha inhibit$.tw.<br>60. PKC-alpha antagonis$.tw.<br>61. Ruboxistaurin.tw.<br>62. C-C chemokine receptor type 2 inhibit$.tw.<br>63. C-C chemokine receptor type 2 antagonis$.tw.<br>64. C-C chemokine ligand type 2 inhibit$.tw.<br>65. C-C chemokine ligand type 2 antagonis$.tw.<br>66. (CC$ adj4 antagonis$).tw.<br>67. (CC$ adj4 inhibit$).tw.<br>68. (CC$ adj3 antagonis$).tw.<br>69. (CC$ adj3 inhibit$).tw.<br>70. Bindarit.tw.<br>71. CCX140-B.tw.<br>72. Darapladib.tw.<br>73. Phospholipase A2 inhibit$.tw.<br>74. Phospholipase A2 antagonis$.tw.<br>75. PLA2 inhibit$.tw.<br>76. PLA2 antagonis$.tw.<br>77. 19 OR 20 OR 21 OR 22 OR 23 OR 24 OR 25 OR 26 OR 27 OR 28 OR 29 OR 30 OR 31 OR 32 OR 33 OR 34 OR 35 OR 36 OR 37 OR 38 OR 39 OR 40 OR 41 OR 42 OR 43 OR 44 OR 45 OR 46	43. Baricitinib:ti,ab,kw<br>44. Tofacitinib:ti,ab,kw<br>45. Upadacitinib:ti,ab,kw<br>46. Nuclear factor erythroid 2-related factor 2 activat*:ti,ab,kw<br>47. NRF 2 activat*:ti,ab,kw<br>48. NRF2 activat*:ti,ab,kw<br>49. Bardoxolone:ti,ab,kw<br>50. ASK 1 inhibit*:ti,ab,kw<br>51. ASK1 inhibit*:ti,ab,kw<br>52. ASK 1 antagonis*:ti,ab,kw<br>53. ASK1 antagonis*:ti,ab,kw<br>54. Apoptosis signal-regulating kinase 1 inhibit*:ti,ab,kw<br>55. Apoptosis signal-regulating kinase 1 antagonis*:ti,ab,kw<br>56. Selonsertib:ti,ab,kw<br>57. Protein kinase C alpha inhibit*:ti,ab,kw<br>58. Protein kinase C alpha antagonis*:ti,ab,kw<br>59. PKC-alpha inhibit*:ti,ab,kw<br>60. PKC-alpha antagonis*:ti,ab,kw<br>61. Ruboxistaurin:ti,ab,kw<br>62. C?C chemokine receptor type 2 inhibit*:ti,ab,kw<br>63. C?C chemokine receptor type 2 antagonis*:ti,ab,kw<br>64. C?C chemokine ligand type 2 inhibit*:ti,ab,kw<br>65. C?C chemokine ligand type 2 antagonis*:ti,ab,kw<br>66. (CC* antagonis*):ti,ab,kw<br>67. (CC* inhibit*):ti,ab,kw<br>68. Bindarit:ti,ab,kw<br>69. CCX140?B:ti,ab,kw<br>70. Darapladib:ti,ab,kw<br>71. Phospholipase A2 inhibit*:ti,ab,kw<br>72. Phospholipase A2 antagonis*:ti,ab,kw<br>73. PLA2 inhibit*:ti,ab,kw<br>74. PLA2 antagonis*:ti,ab,kw<br>75. 19 OR 20 OR 21 OR 22 OR 23 OR 24 OR 25 OR 26 OR 27 OR 28 OR 29 OR 30 OR 31 OR 32 OR 33 OR 34 OR 35 OR 36 OR 37 OR 38 OR 39 OR 40 OR 41 OR 42 OR 43 OR 44 OR 45 OR 46 OR 47 OR 48 OR 49 OR 50 OR 51 OR

OR 47 OR 48 OR 49 OR 50 OR 51 OR 52 OR 53 OR 54 OR 55 OR 56 OR 57 OR 58 OR 59 OR 60 OR 61 OR 62 OR 63 OR 64 OR 65 OR 66 OR 67 OR 68 OR 69 OR 70 OR 71 OR 72 OR 73 OR 74 OR 75 OR 76<br>78. 8 AND 18 AND 77	52 OR 53 OR 54 OR 55 OR 56 OR 57 OR 58 OR 59 OR 60 OR 61 OR 62 OR 63 OR 64 OR 65 OR 66 OR 67 OR 68 OR 69 OR 70 OR 71 OR 72 OR 73 OR 74<br>76. 8 AND 18 AND 75

Figure S1: Cochrane Risk of Bias 2 (RoB2) Tool in the assessment of bias across included studies.

Intention-to-treat	Unique ID	Study ID	Experimental	Comparator	Outcome	Weight	D1	D2	D3	D4	D5	Overall
	De Zeeuw 2013	NA	Bardoxolone	Placebo	NA	1	+	+	!	+	+	!
	De Zeeuw 2015	NA	CCX-140B	Placebo	NA	1	+	+	+	+	+	+
	Knebelmann 201	NA	Bardoxolone	Placebo	NA	1	!	+	+	+	+	!
	Nidorf 2020	NA	Colchicine	Placebo	NA	1	+	+	+	+	+	+
	Ridker 2017	NA	Canakinumab	Placebo	NA	1	+	+	+	+	+	+
	Ridker 2019	NA	Methotrexate	Plaxcebo	NA	1	+	+	+	+	!	!
	Wang 2021	NA	Colchicine	Placebo	NA	1	+	+	+	+	!	!
	SOLID-TIMI 52	NA	Darapladib	Placebo	NA	1	+	+	+	+	!	!
	STABILITY	NA	Darapladib	Placebo	NA	1	+	+	+	+	!	!

+	Low risk
!	Some concerns
-	High risk
D1	Randomisation process
D2	Deviations from the intended interventions
D3	Missing outcome data
D4	Measurement of the outcome
D5	Selection of the reported result

Figure S2: PRISMA diagram.

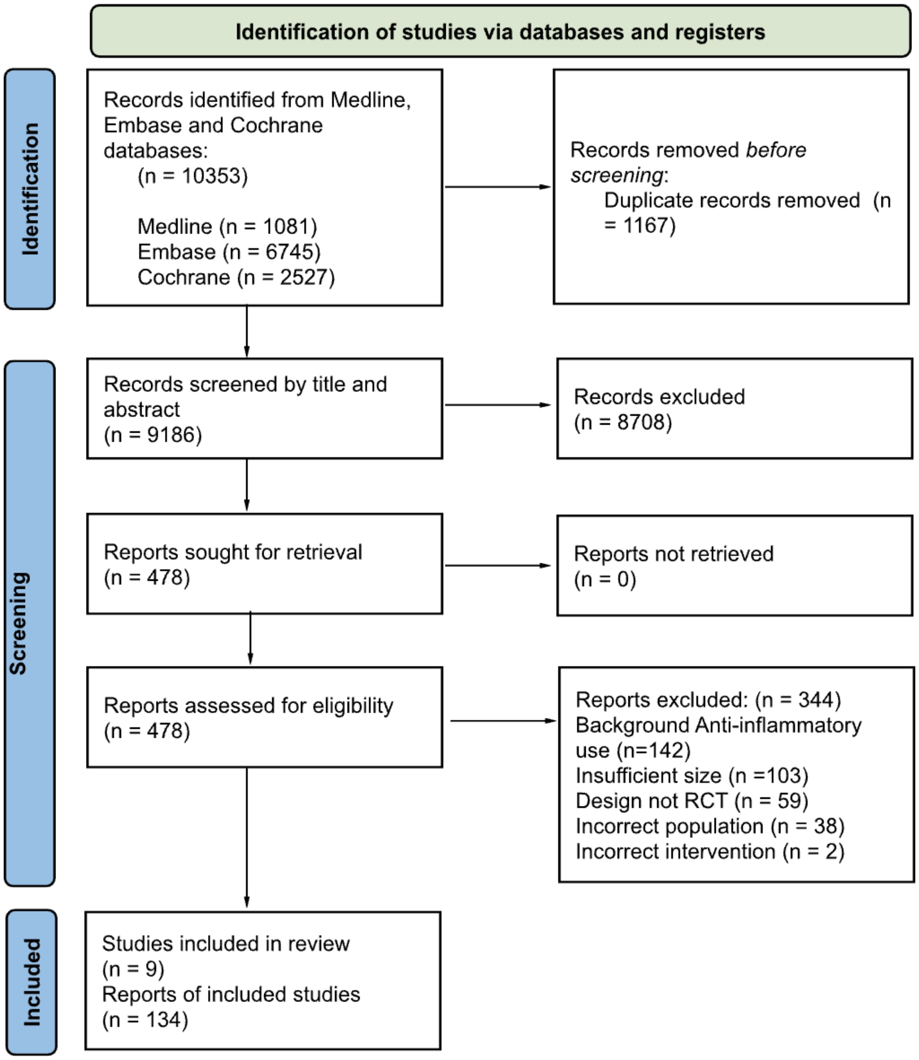

Figure S3: Effect of anti-inflammatories on stroke in a renal population

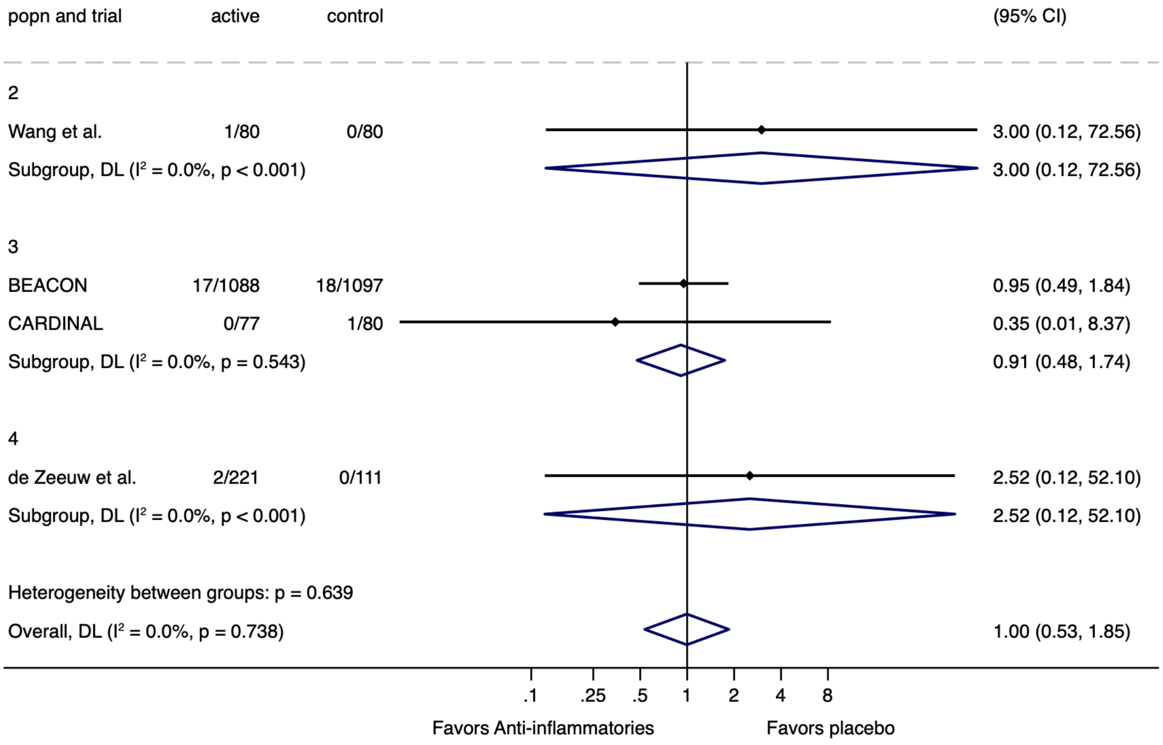

Figure S4: Effect of anti-inflammatories on cardiovascular death in a CKD population

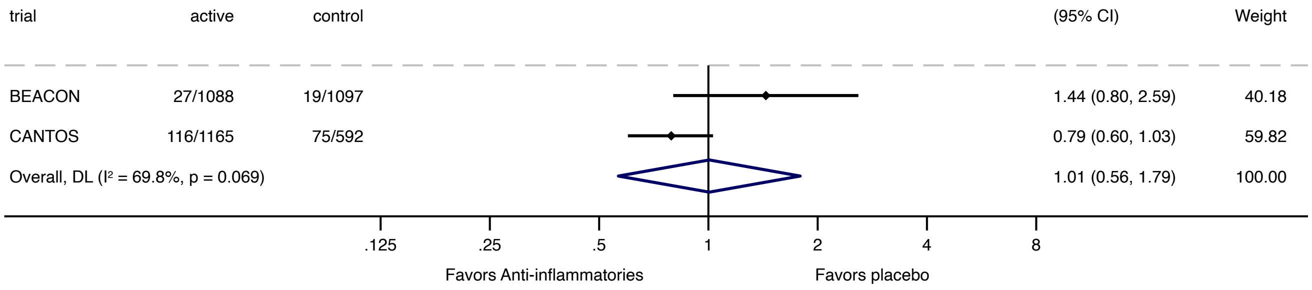

Figure S5: Effect of anti-inflammatories on all-cause mortality in a CKD population

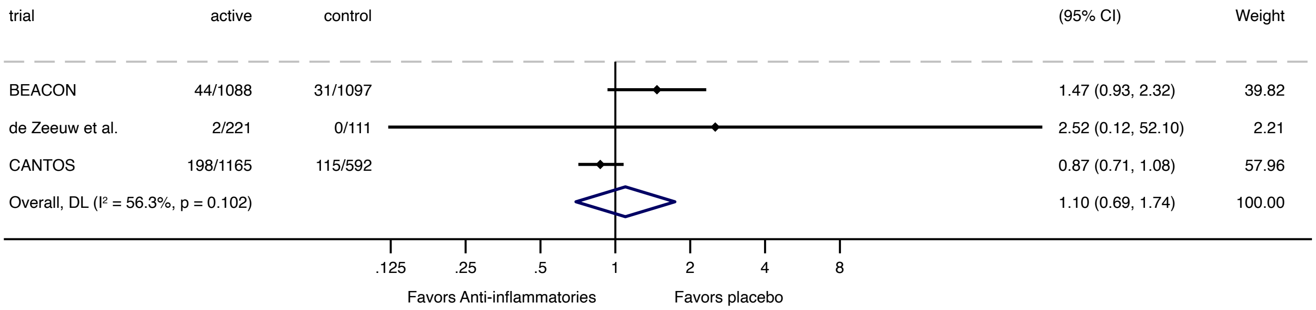

Supplement: sfaf001_Supplemental_File [file sfaf001_Supplemental_File.pdf]
